# An Alzheimer’s disease-associated common regulatory variant in a *PTK2B* intron alters microglial function

**DOI:** 10.1016/j.isci.2026.115688

**Published:** 2026-04-09

**Authors:** Erica Bello, Kathleen Long, Sho Iwama, Juliette Steer, Sarah E. Cooper, Kaur Alasoo, Natsuhiko Kumasaka, Jeremy Schwartzentruber, Nikolaos I. Panousis, Andrew R. Bassett

**Affiliations:** 1 Wellcome Sanger Institute, Wellcome Genome Campus, Hinxton, Cambridge CB10 1SA, UK; 2 OpenTargets, Wellcome Genome Campus, Hinxton, Cambridge CB10 1SA, UK; 3 Milner Therapeutics Institute, Jeffrey Cheah Biomedical Centre, University of Cambridge, Cambridge CB2 0AW, UK; 4 Institute of Computer Science, University of Tartu, Tartu, Estonia; 5 National Center for Child Health and Development, Tokyo, Japan

**Keywords:** Neuroscience, stem cells research, transcriptomics

## Abstract

Identifying and functionally validating the causal variants at genome-wide association study (GWAS) loci is very challenging and has only been achieved for very few variants. Here, we validate a single-nucleotide polymorphism (SNP) associated with increased Alzheimer’s disease (AD) risk in an intronic enhancer of *PTK2B*, by engineering it into human induced pluripotent stem cells (hiPSCs). Upon differentiation to macrophages and microglia, the variant increases chromatin accessibility at the enhancer and binding of transcription factor CEBPB but causes only subtle effects on *PTK2B* or *CLU* expression. Nonetheless, this variant affects both the transcriptome and phenotype of the cells: interferon gamma-responsive genes are downregulated, secreted chemokine levels are reduced, and microglial chemotaxis is affected. We propose the variant acts by altering microglial reactivity, consistent with the established role of these cells in AD progression. This work demonstrates the power of isogenic hiPSC models for functionally validating GWAS-identified common regulatory variants.

## Highlights

• AD risk SNP rs28834970 in *PTK2B* intron increases chromatin accessibility in microglia
• Risk allele alters CEBPB binding and global gene expression, including chemokines
• rs28834970 decreases chemokine secretion and affects microglia chemotaxis

## Graphical abstract

**Figure:**
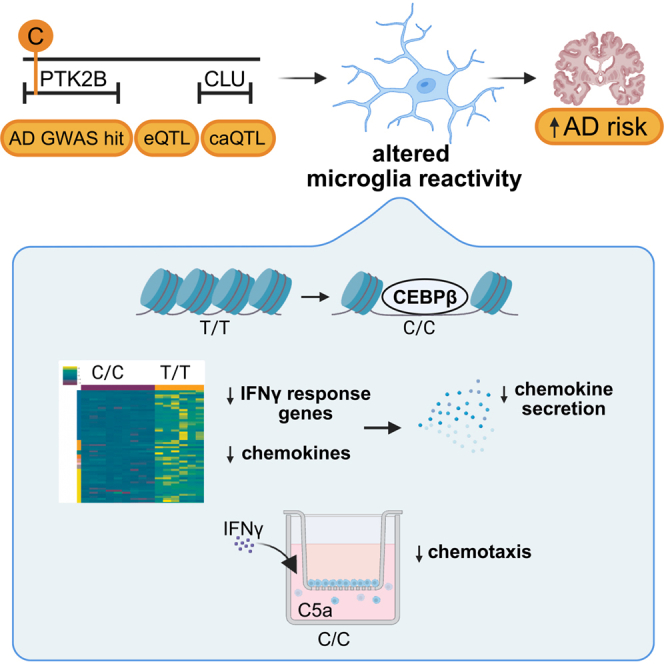

## Introduction

Alzheimer’s disease (AD) is the most common form of dementia, and has a strong genetic component estimated to be 60–80%.^1^ AD is characterized by the presence of β-amyloid-containing plaques (Aβ) and intracellular neurofibrillary tangles of hyperphosphorylated tau, accompanied by neuroinflammation, neuronal, and synapse loss.^2^ Multiple genome-wide association studies (GWASs) have collectively identified more than 80 genetic loci associated with disease,^3^^,^^4^^,^^5^^,^^6^^,^^7^ the vast majority of which do not affect protein-coding sequence. Due to linkage disequilibrium at these loci, it is difficult to identify the causal variants. Multiple computational methods have been developed to prioritize variants that cause disease, including statistical fine mapping, co-localization with expression or chromatin accessibility quantitative trait loci (eQTL, caQTL), overlap with enhancer regions identified by, for example, the assay for transposase accessible chromatin (ATAC) or transcription factor binding in disease-relevant cell types. However, none of these methods is able to unambiguously assign causal variants, and, given the very small number of truly validated variants, it is difficult to benchmark their effectiveness. Experimental methods have also been developed to identify or prioritize variants, such as massively parallel reporter assays (MPRA^8^) and genome engineering-based interrogation of enhancers (GenIE,^9^ GenIE-ATAC^10^), but these are limited to understanding the effects of variants on transcription or chromatin accessibility and do not directly demonstrate the downstream effects of these polymorphisms. Genome engineering of isogenic human cell models, such as differentiated human induced pluripotent stem cells (hiPSCs) differing in a single variant, provides an attractive means for understanding the role of specific single nucleotide polymorphism (SNPs) on cell function and has been successful in understanding the role of strong-effect familial mutations.^11^^,^^12^^,^^13^ However, such systems have yet to be successfully applied to more than a handful of common genetic variants,^14^^,^^15^^,^^16^ and their effects are often confounded by variation between edited clones and during differentiation.^17^

Neuroinflammation has been shown to be a crucial factor in many neurodegenerative disorders, especially AD.^18^ Microglia are resident immune cells of the central nervous system and perform various functions under both homeostatic and disease conditions.^19^ They play important roles in synaptic remodeling, synaptic pruning, and phagocytosis of dead neurons.^20^ Microglia have been implicated in AD by human genetics studies because of the specific expression of AD-related genes in these cells,^21^^,^^22^^,^^23^ colocalization of microglial eQTLs with AD GWAS hits,^24^^,^^25^ and implication of rare causal variants in microglial-specific genes, such as TREM2^26^ and CD33.^26^^,^^27^ Nevertheless, the exact role of microglia in AD pathogenesis is still unclear. Aβ and tau aggregates are able to activate microglia, stimulate cytokines and chemokine production, and elicit an inflammatory response that can have two functions: It can be neuroprotective by increasing Aβ or tau clearance, but it can also contribute to Aβ and tau production and spreading and induce neurodegeneration and synaptic loss.^28^^,^^29^ Microglia are known to be involved in the clearance of aggregated proteins such as Aβ,^30^ and accumulation of extracellular Aβ is accelerated by insufficient microglial activation or phagocytic capacity.^31^ However, the depletion of microglia in amyloid precursor protein-transgenic mouse models was found to have little effect on plaque formation or maintenance. Microglia have also been found to potentially contribute to tauopathy by spreading tau protein via exosomes across brain regions^32^ as well as contributing to its uptake and degradation.^29^ Moreover, sustained microglia activation appears to contribute to neurodegeneration by impacting synaptic plasticity and causing neuronal damage.^33^^,^^34^ As well as microglia, other myeloid cell types, such as infiltrating monocytes,^35^^,^^36^ choroid plexus^37^ and perivascular macrophages,^38^^,^^39^^,^^40^ have also been found to play a role in AD pathogenesis. Circulating monocytes can infiltrate the brain and target cerebral Aβ deposits to reduce plaque load,^41^^,^^42^^,^^43^ and depletion of perivascular macrophages in the TgCRND8 mouse model of AD significantly increases vascular Aβ levels.^44^ Conversely, β amyloid deposition in cortical blood vessels is reduced by the stimulation of perivascular macrophage turnover.^44^ Moreover, studies have shown that macrophages isolated from individuals with AD are poorly phagocytic for Aβ, more susceptible to apoptosis, and have impaired chemotaxis.^45^^,^^46^

Chemokines are produced by several cells in the central nervous system, such as neurons, astrocytes, immune cells, and particularly microglia. They are known to recruit peripheral immune cells through the blood-brain barrier as well as activate brain resident macrophages and microglia.^47^ Elevated levels of several chemokines have been found in plasma, cerebrospinal fluid, and brain tissue of patients with AD.^48^ Proinflammatory chemokines are believed to contribute to the neuroinflammation and chronic activation of microglia and macrophages in late and symptomatic stages of AD^47^ and may constitute a useful biomarker to monitor disease progression.^49^ However, chemokine-mediated microglia recruitment and phagocytosis aid the clearance of Aβ oligomers, and the knockout of some chemokines, such as CCL2 (and CCR2 receptor), in mouse models of AD results in the accumulation of intracellular soluble Aβ oligomers and the decline of cognitive function.^50^^,^^51^^,^^52^ Aβ can stimulate human monocytes and microglia to produce chemokines, such as CXCL8, CCL2, and CCL3, which also play a role in leukocyte migration to sites of inflammation.^53^

PTK2B is a non-receptor tyrosine kinase that is expressed in neurons, microglia, astrocytes, monocytes, and tissue-resident macrophages, and has diverse physiological and pathological roles. These include cell adhesion, cell migration, inflammatory responses, tumor invasiveness,^54^ neuronal development, and plasticity.^55^^,^^56^ PTK2B is a member of the focal adhesion kinase (FAK) subfamily, and after autophosphorylation, it recruits Src-family kinases^57^ and interacts with multiple partners to activate various downstream signaling pathways, such as the MAPK/ERK pathway.^55^ In neurons, PTK2B is implicated in synaptic plasticity^58^ and neurite outgrowth.^59^ In non-neuronal cells, such as osteoclasts,^60^ macrophages,^61^^,^^62^^,^^63^ and monocytes,^64^ PTK2B is involved in the cell migration and regulation of the actin cytoskeleton downstream of integrins. In monocytes, the ROS-sensitive calcium channel TRPM2 can activate the PTK2B/ERK pathway, leading to the nuclear translocation of nuclear factor-kB and increased chemokine production.^65^ This pathway is activated by Aβ in microglia, where PTK2B is autophosphorylated and generates a positive feedback loop to further activate TRPM2.^66^ PTK2B has been implicated in AD through a number of GWAS studies.^4^^,^^6^^,^^67^^,^^68^^,^^69^^,^^70^^,^^71^^,^^72^^,^^73^^,^^74^ Evidence suggests that the genetic deletion of Ptk2b rescues a number of Aβ-associated phenotypes, such as memory impairment, synapse loss, and impaired synaptic plasticity in APP/PS1 animal models of AD.^75^ Conversely, the overexpression of Ptk2b has been reported to be protective in 5×FAD mice^76^ and human neuronal systems.^77^ Similarly, data collected from hemizygous PS19 (MAPT P301S, 1N4R) transgenic mice crossed with Ptk2b^−/−^ animals, points to a protective role for Ptk2b against Tau phosphorylation and Tau-induced behavioral deficits.^78^ Thus, several lines of evidence point to PTK2B as an important player in the pathophysiology of AD, but much remains to be learned about the exact role of PTK2B in AD pathogenesis.

Clusterin (CLU) is a glycoprotein that is mainly secreted but also exists intracellularly and is involved in a variety of cellular functions.^79^ Secreted CLU acts as a molecular chaperone, which stabilizes misfolded proteins and can prevent protein aggregation.^80^ CLU is also involved in modulating inflammatory proteins as well as proteins involved in cell survival and apoptosis.^79^ Cellular stress can lead to an upregulation of CLU, which activates anti-apoptotic pathways and enables cell survival.^81^^,^^82^^,^^83^ CLU is expressed across many tissues, and in the brain, it is expressed in neurons as well as astrocytes and microglia.^79^ Secreted CLU interacts and can bind Aβ, and evidence suggests that this can alter Aβ aggregation and promote clearance.^81^^,^^84^^,^^85^ These studies point to a neuroprotective role of CLU, other evidence suggests that CLU instead reduces Aβ clearance and increases neurotoxicity.^86^^,^^87^ The *CLU* locus is the third greatest genetic risk factor for late-onset AD, and several SNPs in this gene alter AD susceptibility.^79^ CLU is upregulated in the hippocampus, cortex, and CSF of patients with AD, and it colocalizes with Aβ.^81^^,^^88^

AD GWAS studies have identified a locus on chromosome 8 that contains two independent association signals, one over *PTK2B* and one over *CLU*.^67^ Mapping of chromosomal interactions at the *PTK2B* locus in microglia has shown that enhancers harboring AD-risk variants interact with active promoters of both *PTK2B* and *CLU*^89^ but further investigation is needed to identify causal variants at this locus and their putative target genes.

## Results

### Prioritization of a putative Alzheimer’s disease variant at the *PTK2B* locus

**Figure 1:**
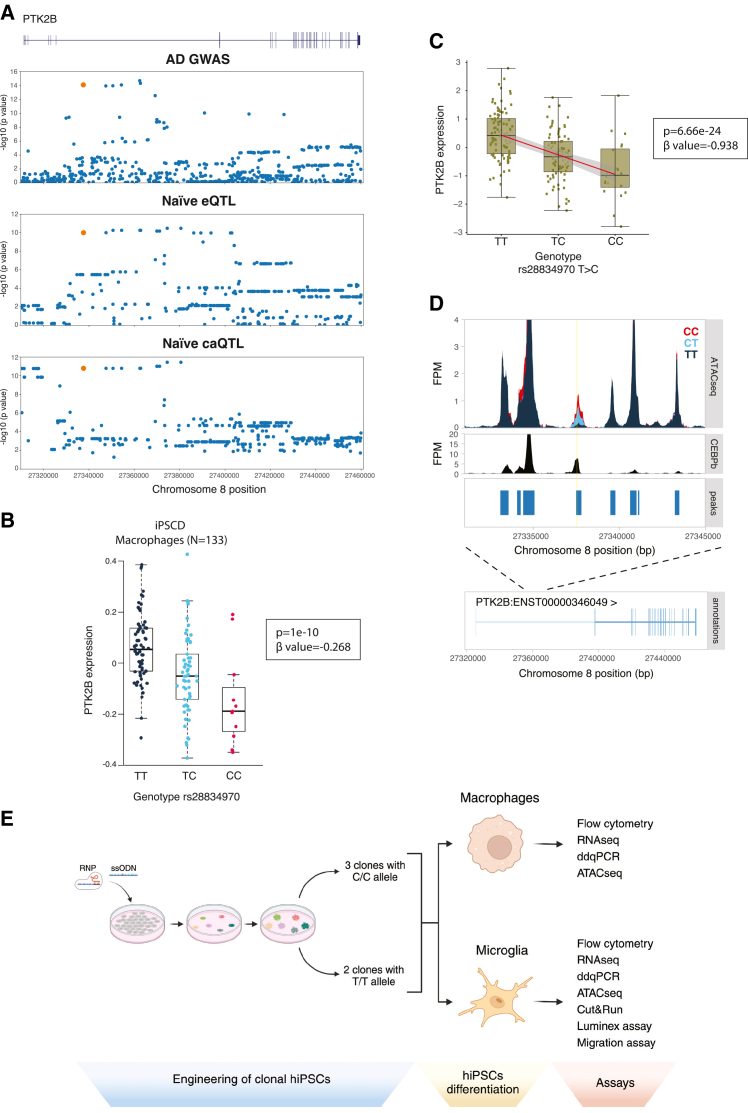
The rs28834970 variant in PTK2B is predicted to modulate an intronic enhancer (A) *PTK2B* gene structure and Manhattan plots for Alzheimer’s disease (AD) GWAS hits (top), eQTL in naive hiPSC-derived macrophages (second), and caQTL in naive hiPSC-derived macrophages (bottom). P-values replotted from.^90^ (B) Boxplots show the median expression of the *PTK2B* gene and interquartile range stratified by the rs28834970 genotype in hiPSC-derived macrophages. Data replotted from.^25^^,^^90^ The y axis shows normalized expression levels (log TPM value), and each dot shows the expression level of a single sample. The permutation p-value obtained via beta approximation is shown. (C) Boxplots show the median expression of the *PTK2B* gene and interquartile range stratified by the rs28834970 genotype in naive hiPSC-derived macrophages. Data replotted from.^91^ The y axis shows normalized expression levels (log TPM value), and each dot shows the expression level of a single sample. The permutation *p*-value obtained via beta approximation is shown. (D) ATAC-seq fragment coverage in hiPSC-derived macrophages stratified by the rs28834970 genotype (top) and CEBPβ ChIP-seq fragment coverage and peaks in primary human macrophages (middle). Data replotted from.^90^ The rs28834970 variant is highlighted in yellow. The bottom shows the structure of the *PTK2B* gene. (E) Overview of the experimental design. Created in BioRender. Bassett, A. (2026) https://BioRender.com/nyfoc26. See also Figure S1.

GWAS have identified two independent genetic associations at the *PTK2B*-*CLU* locus.^67^ The signal over the *PTK2B* gene colocalizes with both an eQTL and a caQTL for *PTK2B* in hiPSC-derived macrophages (Figure 1A).^90^ Re-analysis of publicly available datasets^25^^,^^90^^,^^91^ shows that the risk haplotype is linked to both a downregulation of *PTK2B* gene expression in hiPSC-derived macrophages (Figures 1B and 1C) and an increase in chromatin accessibility at an enhancer within intron 6 of the *PTK2B* gene (Figure 1D). Interestingly, in this case, there is a single variant in the credible set (rs28834970) that lies within the ATAC peak that is changed in accessibility in the caQTL analysis, strongly suggesting that this variant is causal (C being the risk allele). There is some inconsistency in published literature on the directionality of these effects,^25^^,^^90^^,^^91^ but this data has been reanalyzed in conjunction with the authors and in additional hiPSC-derived macrophage datasets, so we are confident of the directionality of these changes described here. This has confirmed that the C allele of rs28834970 is associated with the decreased expression of *PTK2B* in hiPSC-derived macrophages (Figures 1B and 1C), whereas in monocytes and one study of primary microglia, the C allele of rs28834970 is associated with the increased expression of *PTK2B* (Figure S1A).^90^ Of note, this variant was also found to be associated with an increase in *PTK2B* expression in the blood of patients with AD and not in any brain region examined.^92^ In a recent study, which aimed to identify functional GWAS risk variants using allele-specific open chromatin (AsoC) profiling in several hiPSC-derived brain cell models, rs28834970 was identified as an AsoC SNP in iMicroglia but not in any of the other cell types, including neurons.^93^ This evidence suggests that the rs28834970 variant is likely causal and most probably affects specifically myeloid cell phenotypes in the brain but highlights the need for further investigation of the role of this variant in AD pathogenesis.

### Characterization of rs28834970 in hiPSC-derived macrophages and microglia

Given that the *PTK2B* GWAS signal colocalizes with an eQTL/caQTL in hiPSC-derived macrophages, the rs28834970 variant is within a macrophage- and microglia-specific accessible chromatin peak, and it affects allele-specific chromatin accessibility in iMicroglia but not in other cell types,^93^ we decided to focus our investigation on the role of this variant in macrophages and microglia, although we cannot exclude a role in other cell types. We edited the C allele of rs28834970 into a well-characterized hiPSC line (KOLF2_C1) that was homozygous for the T allele at this position (and thus the non-risk haplotype) using CRISPR-mediated homology-directed repair^94^ (Figure S1B and STAR Methods). This allows us to unambiguously study the role of rs28834970 independently of the other variants in linkage disequilibrium. We subsequently analyzed three independent homozygous (C/C) clones and two unedited (T/T) clones, one of which had been through the editing process, and the second was the parental line. These were differentiated in three biological replicates into either macrophages^95^^,^^96^ or microglia,^97^^,^^98^ followed by a number of assays including transcriptomics (RNAseq), chromatin accessibility (ATACseq), and quantitative digital RT-PCR (dd-qRT-PCR) to measure gene expression (Figure 1E). Differentiation was highly reproducible between the two different genotypes by FACS for macrophage markers CD14, CD16, and CD206 (Figure S2A) and microglia markers CD11b and CD45 (Figure S2B). This was further validated by the analysis of macrophage and microglial marker gene expression,^97^^,^^99^ which were mostly consistent with the differentiation trajectory (Figure S2C), including the homeostatic microglial marker P2RY12 that distinguishes between microglia and macrophages and microglial-enriched genes, e.g., TREM2, GPR34, and C1Qa.^97^^,^^99^ Moreover, there did not appear to be genotype-dependent changes in the expression of canonical markers of macrophage or microglial activation (Figure S2D).

### rs28834970 introduces a novel CEBPB binding site that increases chromatin accessibility at an intronic enhancer in hiPSC-derived macrophages and microglia

**Figure 2:**
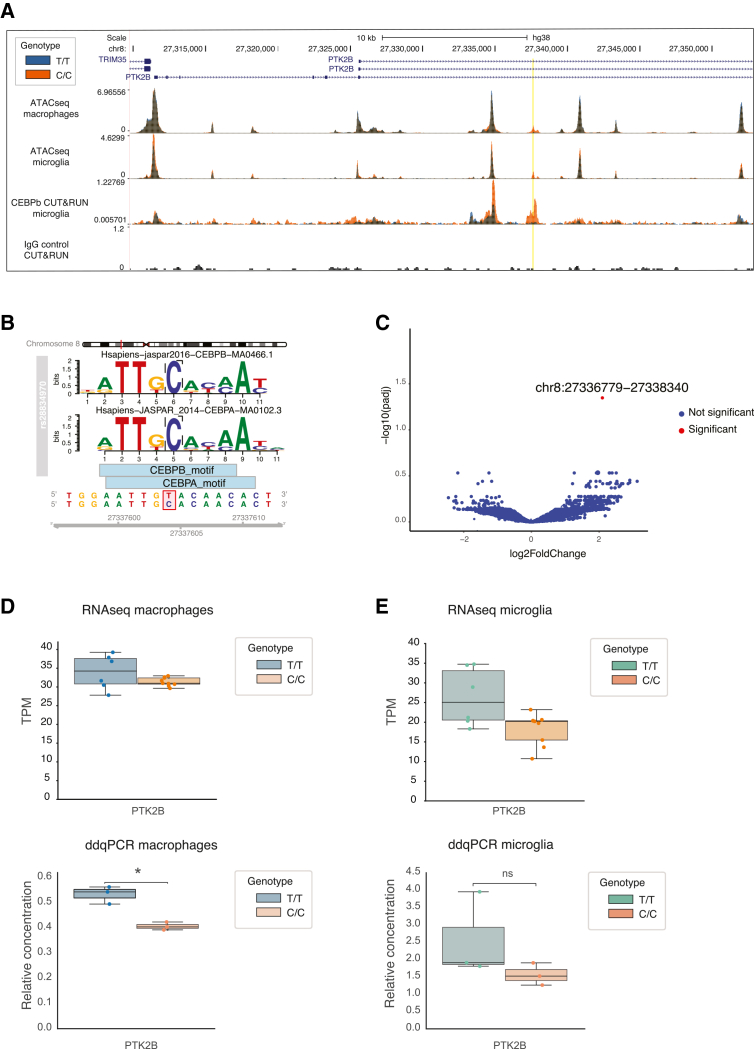
Isogenic edited cell lines show that rs28834970 affects chromatin accessibility and CEBPβ binding at the intronic enhancer in hiPSC-derived macrophages and microglia, but only modestly decreases *PTK2B* expression (A) Coverage tracks of cells with T/T (orange) and C/C (blue) allele at rs28834970. The tracks are: ATAC-seq in macrophages (upper track), ATAC-seq in microglia (second track), CUT&RUN for CEBPβ in microglia (third track), and IgG negative control for CUT&RUN (bottom track). Normalized read counts per basepair are plotted, and the rs28834970 variant in *PTK2B* intron is highlighted in yellow. (B) Effect of rs28834970 SNP on transcription factor binding probability. Position probability matrices are shown for CEBPβ (top) and CEBPα (middle) as well as the position of the binding motifs on the *PTK2B* sequence (bottom). The plot was generated using motifbreakerR.^100^ (C) Volcano plot shows fold changes of CEBPβ binding between microglia harboring the C/C and the T/T allele for all detectable peaks, measured by CUT&RUN. Blue dots indicate peaks with no significant change, while red dots represent significantly increased peaks (log2FC > 1 and adjusted *p* < 0.05). The only significantly increased CEBPβ peak found spans the rs28834970 variant in the *PTK2B* intron. (D) Boxplots show *PTK2B* expression in macrophages harboring the T/T or C/C allele at rs28834970 measured by RNA-seq (top) and droplet digital q-PCR (ddqPCR) (bottom). Top plot: TPM = transcripts per million. Bottom plot: Relative concentration = copies/ul relative to housekeeping gene (SDHA), *n* = 3 and ∗*p* < 0.05, unpaired *t* test. Boxplot shows median expression and interquartile range. (E) Boxplots show *PTK2B* expression in microglia harboring the T/T or C/C allele at rs28834970 measured by RNA-seq (top) and droplet digital q-PCR (ddqPCR) (bottom). Top plot: TPM = transcripts per million. Bottom plot: Relative concentration = copies/ul relative to housekeeping gene (TBP), *n* = 3, and ns = non-significant, unpaired *t* test. Boxplot shows median expression and interquartile range. See also Figure S2 and Tables S1, S2, S3, S4, and S6.

We analyzed the effect of the rs28834970 allele on the chromatin accessibility and function of the intronic enhancer element. ATACseq analysis showed that the C allele created a novel region of accessible chromatin in both macrophages and microglia (Figure 2A), which was consistent with the expectations from the caQTL analysis in hiPSC-derived macrophages (Figure 1D). The analysis of chromatin accessibility within a 600 kb window around the SNP (Figure S3A) showed that there was no change in most peaks, and the only statistically significant change consistent between macrophages and microglia was the new peak over rs28834970 (macrophages: *p* = 1.65e−16 log2FC = 2.71, microglia: *p* = 4.19e−12 log2FC = 2.77) (Figure S3B). Peaks within the *CHRNA2* and *CLU* genes were significantly reduced in microglia, and an intergenic peak was increased in macrophages, but these were not consistent between the two cell types, or with the expression changes of these genes (Figure S3B; Table S1).

Further analysis of the sequence around rs28834970 showed that the C allele increased the probability of binding of transcription factors CEBPA and CEBPB (Figure 2B). We thus analyzed CEBPB binding to DNA by CUT&RUN, which showed an increased binding for the C allele of rs28834970 (*p* = 0.04 log2FC = 2.09) (Figure 2A), consistent with CEBPB binding being responsible for the change in chromatin accessibility. Indeed, in a genome-wide analysis, this was the only region that showed a significant change in CEBPb binding between the C/C and T/T alleles at rs28834970 (Figure 2C; Table S2).

We next analyzed the effect of rs28834970 on the expression of *PTK2B* and the other genes within a 600 kb window around this variant (Figure S3A). *CHRNA2* and *EPHX2* are not expressed in macrophages or microglia and were therefore not investigated. Both RNAseq and digital droplet qPCR (dd-qPCR) analysis showed that the expression of *PTK2B* was only modestly reduced by the introduction of the C allele in macrophages (RNAseq: *p* = 0.019 and log2FC = −0.2, ddqPCR: *p* = 0.02 and log2FC = −0.39) (Figure 2D) and microglia (RNAseq: *p* = 0.06 log2FC = −0.57, ddqPCR: *p* = 0.29 and log2FC = −0.71) (Figure 2E) and it was only borderline significant with ddqPCR in macrophages and not microglia. However, this effect is consistent in direction between RNAseq and ddqPCR and across microglia and macrophages, and it is in line with the effect of the eQTL (Figures 1B and 1C). The effect on *PTK2B* expression was the most consistent change in microglia and macrophages within the window around rs28834970, but there was additionally a borderline significant upregulation of *CLU* expression specifically in macrophages (RNAseq: log2FC = 0.72, p = 0.03), which was not observed in microglia (Figure S3C). This suggests that there may be an effect of the rs28834970 enhancer on *PTK2B* and *CLU* expression, but it is not consistently significant between experiments. This could be due to the small effect size of such common regulatory variants, which makes it difficult to detect in these assays, or to other mutations in the same haplotype contributing further to expression changes in this locus. We also analyzed changes in splicing of the *PTK2B* gene and did not detect any significant changes in splicing of the exons near the variant (Figure S3D).

Taken together, these results validate that rs28834970 within the *PTK2B* locus is a regulatory variant that acts through introducing a novel CEBPB binding site that increases chromatin accessibility at the intronic enhancer. There is a subtle decrease in *PTK2B* expression and an increase in CLU expression caused by rs28834970, but this is overall not significant, so it is difficult to be sure whether the effect of rs28834970 is mediated through *PTK2B* or *CLU* expression.

### rs28834970 causes strong effects on the transcriptome, including chemokine expression

**Figure 3:**
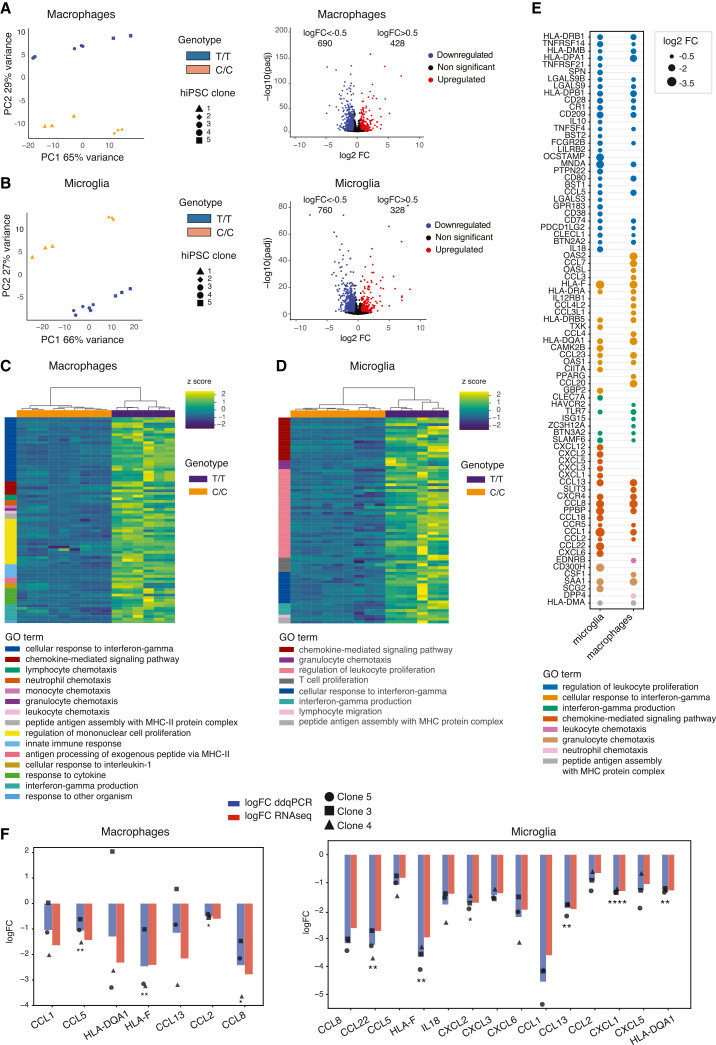
The rs28834970 variant affects global gene expression in hiPSC-derived macrophages and microglia (A) Results of differential expression analysis of macrophages harboring the C/C allele versus the corresponding hiPSC-derived cells harboring the T/T allele at rs28834970. Left panels show principal component analysis (PCA) of transcriptomes of cells differentiated from two hiPSC clones with the T/T allele and three clones with the C/C allele. The right panel is a volcano plot showing the log2 fold-change (FC) between cells with the C/C and the T/T allele and corresponding *p* values for all transcribed genes. Significantly upregulated genes (log2FC > 0.5 and adjusted *p* < 0.05) are shown as red dots, significantly downregulated genes (log2FC < −0.5 and adjusted *p* < 0.05) are shown as blue dots, and non-significant genes as black dots. (B) Results of differential expression analysis of microglia harboring the C/C allele versus the corresponding hiPSC-derived cells harboring the T/T allele at rs28834970. Left panels show principal component analysis (PCA) of transcriptomes of cells differentiated from two hiPSC clones with the T/T allele and three clones with the C/C allele. The right panel is a volcano plot showing the log2 fold-change (FC) between cells with the C/C and the T/T allele and corresponding *p* values for all transcribed genes. Significantly upregulated genes (log2FC > 0.5 and adjusted *p* < 0.05) are shown as red dots, significantly downregulated genes (log2FC < −0.5 and adjusted *p* < 0.05) are shown as blue dots, and non-significant genes as black dots. (C) Heatmaps show relative expression (z-scores) of DE genes found in gene ontology (GO) analysis in macrophages with the T/T (purple) and the C/C (orange) allele at rs28834970. GO pathways identified are also annotated. (D) Heatmaps show relative expression (z-scores) of DE genes found in gene ontology (GO) analysis in microglia with the T/T (purple) and the C/C (orange) allele at rs28834970. GO pathways identified are also annotated. (E) Dot plot shows log2FC of downregulated genes belonging to the top eight GO pathways in macrophages and microglia. (F) Bar graph shows the mean log2FC of downregulated chemokines and HLA genes in macrophages (left) and microglia (right) per clone measured by RNAseq (blue) and ddqPCR (orange). ddqPCR: *n* = 3, ∗*p* < 0.05, ∗∗*p* < 0.01, ∗∗∗*p* < 0.001, and ∗∗∗∗*p* < 0.0001, an unpaired *t* test. Individual hiPSC clones are indicated by different shapes. See also Figures S3 and S4; Tables S3, S4, and S6.

We next analyzed the effects of editing rs28834970 on genome-wide gene expression in macrophages and microglia. In both cell types, principal component analysis showed that although there was a strong clone and batch effect, the two genotypes were well separated by the second principal component (Figures 3A and 3B). Similar results were seen with the analysis of global chromatin accessibility, where genotypes were separated by the second principal component (Figure S4A). The analysis of the differentially expressed genes between genotypes showed 690 downregulated and 428 upregulated genes in macrophages (Figure 3A), and 760 downregulated and 328 upregulated in microglia (Figure 3B; Table S3). Importantly, there was a significant (*p* < 0.0001) overlap in the differentially expressed genes between the two cell types (Figure S4B), further corroborating that we were identifying true effects of the genotype, not simply variation between edited clones or differentiation artifacts.

We performed gene set enrichment analysis of the differentially expressed genes relative to all genes expressed in the respective cell type. This showed that a number of gene ontology (GO) pathways relevant to microglial or macrophage function were enriched in the downregulated gene set, including chemokine-mediated signaling, cellular response to interferon gamma (IFNγ), and migration and chemotaxis (Figures 3C and 3D). These pathways were frequently overlapping between microglia and macrophages (Figure S4C), again supporting that these changes were genotype-dependent. Conversely, there were no obviously relevant GO enrichments in the upregulated gene set (Figure S4D), which may be due to the smaller number of genes analyzed.

Further analysis of the downregulated genes confirmed that there was considerable overlap between microglia and macrophages across the different GO categories (Figure 3E). Of note, we found that the macrophage receptor with collagenous structure (*MARCO*) gene, was among the top 3 downregulated genes in both microglia and macrophages with the C/C allele (Figure S4E). MARCO is a putative Aβ receptor that triggers uptake and downstream signaling in microglia.^101^^,^^102^ Given their role in microglial and macrophage function, we focused on the downregulation of chemokines, MHC complex proteins, and surface receptors and validated their expression by dd-qPCR (Table S4). All of the chosen genes showed some level of downregulation as expected in microglia, and all except one (*HLA-DQA1*) were downregulated in macrophages (Figure 3F), with effect sizes generally consistent between dd-qPCR and RNAseq.

### rs28834970 causes changes in chemokine release and migration

**Figure 4:**
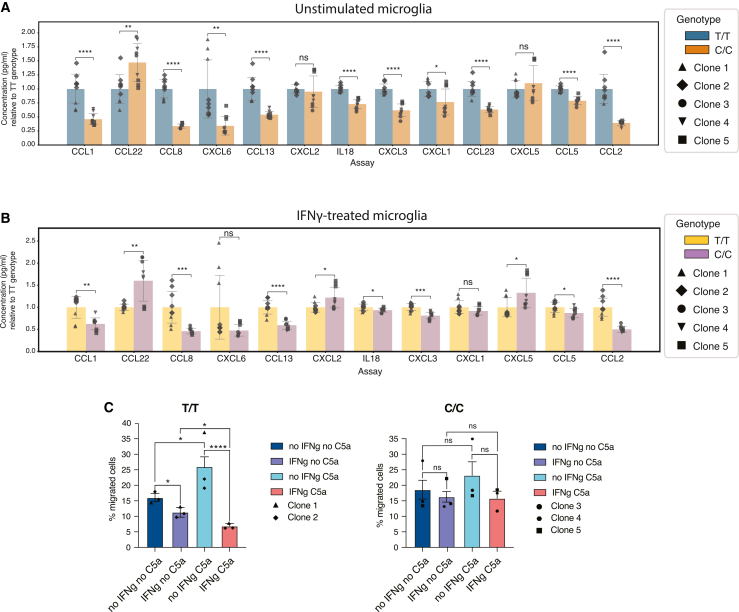
The rs28834970 variant causes changes in chemokine production and migration in hiPSC-derived microglia (A) Concentration of downregulated chemokines in unstimulated microglia with the C/C allele at rs28834970 relative to the concentration in clones with the T/T allele, measured by Luminex assay. Bars represent the mean ± SEM of independent biological replicates (*n* = 3) of each of the five differentiated hiPSC clones, and dots represent technical replicates per clone. ns = non-significant, ∗*p* < 0.05, ∗∗*p* < 0.01, ∗∗∗*p* < 0.001, and ∗∗∗∗*p* < 0.0001, an unpaired *t* test. (B) Concentration of downregulated chemokines in microglia with the C/C allele at rs28834970 relative to the concentration in clones with the T/T allele, stimulated with IFNγ. Bars represent the mean ± SEM of independent biological replicates (*n* = 3) of each of the five differentiated hiPSC clones, and dots represent technical replicates per clone. ns = non-significant, ∗*p* < 0.05, ∗∗*p* < 0.01, ∗∗∗*p* < 0.001, and ∗∗∗∗*p* < 0.0001, an unpaired *t* test. (C) Bar graph shows the percentage of migrated microglia harboring the T/T allele (left) or the C/C allele (right), either unstimulated or stimulated with IFNγ, and with or without the chemoattractant C5a. Bars represent the mean ± SEM of independent biological replicates (*n* = 3) of each of the five differentiated hiPSC clones, and dots represent the average of the three replicates of each clone. ns = non-significant, ∗*p* < 0.05, ∗∗*p* < 0.01, and ∗∗∗*p* < 0.001, unpaired *t* test. See also Figure S5 and Table S5.

Guided by the consistent effects on chemokine and migration-related gene expression in both macrophages and microglia, we next analyzed the release of 13 chemokines using a multiplexed assay on microglial cell supernatants from all differentiated clones. In unstimulated cells, we saw a significant reduction in the production of 11 of the 13 chemokines tested with the C/C allele at rs28834970, consistent with the results of the transcriptomic analysis (Figure 4A). CCL22 was unexpectedly upregulated, and CXCL2 and CXCL5 were unchanged (Figure 4A), which may be a result of the different readouts measured by the two assays (mRNA vs. secreted protein).

Next, we aimed to evaluate the production of these chemokines in stimulated microglia. We treated microglia with IFNγ and lipopolysaccharide (LPS) and analyzed whether chemokine production was altered in stimulated microglia. As expected, both stimulations increased production of most (10 of 12) of the chemokines (Figures S5A and S5B). CCL1 production was increased by LPS stimulation in microglia harboring the C/C allele but not in microglia with the T/T allele (Figure S5B). Similar to what was observed in unstimulated cells, production of most of the chemokines (10 of 12) was reduced in the C/C allele compared to the T/T allele at rs28834970 when microglia were stimulated with IFNγ, but the magnitude of the reduction was reduced (Figure 4B). A similar response was seen upon stimulation with LPS (Figure S5C). Taken together, these data suggest that the C/C allele results in a lower basal chemokine release, and in the presence of stimuli such as IFNγ, this reduction becomes less marked (Table S5).

We further investigated the effect of rs28834970 on migration and chemotaxis of microglia differentiated from all hiPSC clones, using a transwell assay in the presence or absence of IFNγ and with or without a chemoattractant relevant to microglia, complement 5a (C5a). Microglia harboring the unedited (T/T) allele showed chemotaxis toward C5a that was inhibited by IFNγ treatment (Figure 4C). Microglia harboring the C/C alleles at rs28834970 showed no significant chemotaxis toward C5a or inhibition of migration following IFNγ treatment (Figure 4C). Taken together, these data suggest that microglia harboring the C/C allele are less sensitive to the chemoattractant C5a and to the inhibitory effect of IFNγ on migration.

In summary, we show that the C allele of rs28834970 lowers chemokine release by microglia, reduces chemotaxis toward C5a and alters their response to IFNγ stimulation, perhaps by altering microglia reactivity.

## Discussion

One of the major challenges in interpreting GWAS is to understand the genes and causal variants underlying the genetic association with disease. The use of isogenic cell lines generated by CRISPR in model systems such as hiPSC differentiated cell types offers one way to dissect the role of individual variants in cellular phenotypes independently of other variants in linkage disequilibrium. However, common regulatory variants that are identified in GWAS have rarely been linked to changes in cellular ‘omics or phenotypes. Here, we identify a likely causal variant from comparison of AD GWAS, eQTL, and caQTL studies and engineer this using CRISPR/Cas9 into hiPSCs to generate isogenic cell lines differing in this single variant. We show that the C/C allele of rs28834970 introduces a novel CEBPB binding site that increases chromatin accessibility at an intronic enhancer, consistent with the results of the caQTL analysis in hiPSC-derived macrophages.

Previous eQTL analysis showed that this variant causes a decrease in *PTK2B* expression in monocytes^103^ and macrophages^25^^,^^90^^,^^91^ (Figures 1B and 1C). However, in our study, we observed only a slight downregulation of *PTK2B* in microglia and macrophages expressing the C/C allele. We also found a subtle upregulation of *CLU* expression at borderline significance in macrophages but not in microglia expressing the C/C allele, making it difficult to make a definite conclusion on whether rs28834970 affects the expression of either *PTK2B* or *CLU* or a combination of the two. This could be due to the small effect size, making it challenging to study with a limited number of isogenic cell clones, the contribution of other SNPs within the same risk haplotype, or compensatory regulatory mechanisms that may buffer expression levels. Two recent studies identified SNPs rs1532278 and rs11136000 as causal variants at the CLU locus, which upregulate CLU expression and influence the function of glutaminergic neurons and astrocytes, respectively.^104^^,^^105^ However, since neither of these variants is present in our hiPSC lines nor are they in linkage disequilibrium with rs28834970, they are unlikely to influence *PTK2B* and *CLU* expression in this context.

In spite of the subtle changes in *PTK2B* or *CLU* expression, we found that the C/C allele at rs28834970 caused a large transcriptional response, including the downregulation of chemokines and of genes involved in cellular response to IFNγ. This risk variant also resulted in phenotypic changes, such as a reduction in chemokine release and decreased chemotaxis toward C5a. Taken together, this suggests that rs28834970 is a functional regulatory variant associated with AD risk at this locus.

We propose that rs28834970 causes a change in microglia reactivity, resulting in a different response to stimulations such as IFNγ. This may impact the ability of the microglia to respond to aggregated proteins such as Aβ plaques and cellular damage in the brain, therefore increasing the risk of AD. Indeed, several pro-inflammatory chemokines, such as CCL2, CCL5, and IL18, which are downregulated in microglia with the C/C allele both in the naive state and when stimulated with IFNγ, are produced by microglia in response to Aβ and are important for Aβ-induced microglia chemotaxis.^106^^,^^107^^,^^108^ Even though the overexpression of these chemokines is characteristic of neuroinflammation, correlated with disease progression, and found in late stages of AD, knockout of chemokines, such as CCL2, and chemokine receptors, such as CCR2 and CCR5, in mice is associated with increased Aβ deposition and accumulation.^47^^,^^50^^,^^51^^,^^52^^,^^109^ It has also been found that patients carrying the CCR5Δ32 mutation, which prevents CCR5 surface expression, develop AD at a younger age.^110^ Therefore, we hypothesize that in individuals carrying the C/C (risk) allele of rs28834970, downregulation of these chemokines in macrophages and microglia affects Aβ-induced microglia chemotaxis, leukocyte recruitment, and clearance of Aβ, and may increase the risk of developing symptomatic AD. The decrease in the expression of the putative Aβ receptor *MARCO* in the C/C allele may also contribute to this effect. We believe this study demonstrates the power of isogenic cell lines to identify and validate individual variants, including common regulatory SNPs identified from GWAS.

The integration of polygenic risk scores incorporating noncoding variants such as rs28834970, into clinical assessment protocols could enhance disease forecasting by identifying individuals with altered microglial reactivity patterns before symptom onset or stratifying patients into different functional causes of disease. Furthermore, understanding how risk variants affect the trajectory of microglial and macrophage responses to pathological stimuli such as Aβ aggregation may reveal novel therapeutic strategies, offering opportunities to restore protective immune clearance mechanisms in genetically susceptible populations.

### Limitations of the study

This study has several limitations that should be considered when interpreting the results. A key challenge inherent to the functional validation of common regulatory variants identified by GWAS is their small effect size, which makes detecting statistically significant changes in gene expression and cellular phenotypes difficult with a limited number of isogenic clones. Here, we used three homozygous C/C clones and two T/T clones, and while this design allowed us to identify consistent transcriptomic and phenotypic differences between genotypes, a larger sample size of C/C and T/T clones would increase statistical power and reduce the risk that observed effects reflect clone-to-clone variation rather than the variant itself. Similarly, all clones were derived from a single hiPSC line (KOLF2_C1) derived from a white British male; engineering rs28834970 into additional hiPSC lines from female donors, donors of different ethnicity, and diverse genetic backgrounds would help determine whether the effects observed are generalizable across individuals. Furthermore, while we observed subtle and not always statistically significant changes in *PTK2B* and *CLU* expression in C/C lines, the causal relationship between these modest expression changes and the broader transcriptomic and phenotypic signatures remains unclear. Future experiments involving knockdown and overexpression of *PTK2B* and *CLU* individually and in combination would help establish whether the functional effects of rs28834970 are mediated through altered expression of one or both of these genes, or through alternative mechanisms. Finally, while our study investigates the impact of rs28834970 in microglia and macrophages, this variant’s regulatory mechanisms might also be cell-type specific. PTK2B is expressed in neurons and is important for their function^58^^,^^111^ and we cannot exclude that rs28834970 might impact *PTK2B* expression and function differently in neurons, blood-derived myeloid cells, and other cell types.

## Resource availability

### Lead contact

Further information and requests for resources and reagents should be directed to and will be fulfilled by the lead contact, Andrew Bassett (ab42@sanger.ac.uk) or Erica Bello (eb956@cam.ac.uk).

### Materials availability

Isogenic hiPSC lines generated in this study are available from the lead contact with a completed materials transfer agreement.

### Data and code availability

• Raw sequencing data have been deposited at the European Nucleotide Archive (ENA) and are publicly available as of the date of publication. ATACseq and CUT&RUN data are available under accession number ENA: ERP024123, and RNAseq data under accession number ENA: ERP024128. A description of all sample IDs is provided in Table S6. This paper analyses existing, publicly available data accessible at Zenodo: https://doi.org/10.5281/zenodo.1158560, SynID: syn26207321 and ENA: EGAS00001002268. See also the key resources table.
• All original code has been deposited at Zenodo: https://doi.org/10.5281/zenodo.19360201 and is publicly available as of the date of publication. See also the key resources table.
• Any additional information required to reanalyze the data reported in this paper is available from the lead contact upon request.

## Acknowledgments

This work was funded by OpenTargets [OTAR037] and 10.13039/100004440Wellcome 220540/Z/20/A. For the purpose of Open Access, the author has applied a CC BY public copyright license to any Author Accepted Manuscript version arising from this submission. We thank the Sanger Institute flow cytometry core facility for flow cytometry analysis. We acknowledge Scientific Operations at the Sanger Institute for support in next-generation sequencing and quality control. We thank members of the Bassett lab and Magdalena Strauss for helpful discussions and support, and Marta Perez-Alcantara for supporting computational analysis. The graphical abstract was created with BioRender.com.

## Author contributions

A.B. and E.B. conceived the work and designed experiments, K.L. made the hiPSC isogenic cell lines, E.B., S.I., S.C., and J.S. executed the experiments, E.B., K.A., N.K., J.S., and N.I.P. analyzed data, E.B. and A.B. wrote the manuscript with input from all other authors.

## Declaration of interests

A.B. is a founder of and consultant for EnsoCell Therapeutics. N.I.P. was an employee of GSK at the time the manuscript was submitted. J.S. was an employee of Illumina at the time the manuscript was submitted.

## STAR★Methods

### Key resources table

**Table:** 

REAGENT or RESOURCE	SOURCE	IDENTIFIER
**Antibodies**		

PE-anti human CD45 antibody clone HI30	Biolegend	Cat# 304008; RRID:AB_314396
APC-anti human CD11b antibody clone ICRF44	Biolegend	Cat# 301310; RRID:AB_314162
APC/Cy7-anti human CD16 antibody clone 3G8	Biolegend	Cat# 302018; RRID:AB_314216
APC-anti human CD206 antibody clone 19.2	BD Biosciences	Cat# 550889; RRID:AB_398476
PE-anti human CD14 antibody clone M5E2	BD Biosciences	Cat# 555398; RRID:AB_395798
APC-mouse IgG1 k isotype control clone MOPC-21	BD Biosciences	Cat# 555751; RRID:AB_555751
PE-mouse IgG2A isotype control clone G155-178	BD Biosciences	Cat# 555574; RRID:AB_395953
APC/Cy7-mouse IgG1 k isotype control clone MOPC-21	BD Biosciences	Cat# 400122; RRID:AB_326443
C/EBP beta Antibody (H-7) sc-7962 (CUT&RUN)	Santa Cruz Biotechnology	Cat# sc-7962; RRID:AB_626772
CUTANA Rabbit IgG CUT&RUN Negative Control Antibody	EpiCypher	Cat# 13–0042; RRID:AB_2923178

**Biological samples**		

Mouse Embryonic Fibroblasts (MEFs)	Merck	Cat# PMEF-CFX

**Chemicals, peptides, and recombinant proteins**		

eSpCas9 protein (purified in-house)	Slaymaker et al., 2016 (ref.^100^)	N/A
Recombinant Human FGF (4 ng/mL)	R&D Systems	Cat# 233-FB
rhM-CSF (50 ng/mL, macrophage differentiation)	R&D Systems	Cat# 216-MC
rhIL-3 (25 ng/mL)	R&D Systems	Cat# 203-IL
rhM-CSF (100 ng/mL, macrophage terminal differentiation)	R&D Systems	Cat# 216-MC
rhBMP-4 (50 ng/mL)	Peprotech	Cat# 120-05
rhSCF (20 ng/mL)	Peprotech	Cat# 300-07
rhVEGF (50 ng/mL)	Peprotech	Cat# 100-20
rhGM-CSF (10 ng/mL)	Peprotech	Cat# 300-03
rhIL-34 (100 ng/mL)	Peprotech	Cat# 200-34
E. coli Spike-in DNA (0.5 ng per sample)	EpiCypher	Cat# 18-1401
Recombinant Human IFN-gamma (IFNy)	R&D Systems	Cat# 285-IF
Lipopolysaccharide (LPS) from E. coli	Invitrogen	Cat# 00-4976-93
Recombinant Human Complement C5a	PeproTech	Cat# 300-70-100UG

**Critical commercial assays**		

Illumina Tagment DNA TDE1 Enzyme and Buffer Kit	Illumina	Cat# 20034197
NEBNext Ultra II DNA Library Prep Kit for Illumina	NEB	Cat# E7645
NEBNext Multiplex Oligos for Illumina (Dual Index Primers Set 1)	NEB	Cat# E7600
CUTANA CUT&RUN Kit	EpiCypher	Cat# 14-1048
ProcartaPlex Human Basic Kit	Invitrogen	Cat# EPX010-10000-901
ProcartaPlex Human Simplex analyte beads (panel of 13 chemokines)	Invitrogen	NA

**Deposited data**		

ATACseq and CUT&RUN raw sequencing data	European Nucleotide Archive	ENA: ERP024123
RNAseq raw sequencing data	European Nucleotide Archive	ENA: ERP024128
eQTL and caQTL data in hiPSC-derived macrophages (Alasoo et al., 2018)	Zenodo	ID: 1158560
Microglia eQTL/caQTL data (Young et al., 2021)	AD Knowledge Portal	SynID: syn26207321
hiPSC-macrophage eQTL data (Panousis et al., 2023)	European Nucleotide Archive	ENA: EGAS00001002268

**Experimental models: Cell lines**		

KOLF2_C1 parental hiPSC line (T/T allele at rs28834970)	HipSci project	RRID: CVCL_9S58; https://hpscreg.eu/cell-line/WTSIi018-B-1
Edited hiPSC clone C8 (C/C allele at rs28834970)	This paper	N/A
Edited hiPSC clone C11 (C/C allele at rs28834970)	This paper	N/A
Edited hiPSC clone D3 (C/C allele at rs28834970)	This paper	N/A
Unedited hiPSC clone B11 (T/T allele at rs28834970)	This paper	N/A

**Oligonucleotides**		

sgRNA sequence for rs28834970 editing: AGTGGAATTGTACAACACTG	Synthego	N/A
ssODN repair template for rs28834970 C/C allele engineering: CTGGCAAGACTAATCTACTTTCTATTTTTATGGA TCTGCCTTTTCTGGTCATTCCATATAAGTGGAA TTGCACAACACTGTGGCCTTTCGCGACGGCT GCTTTCACTTAGCACAATGTTTTGAAACTTCC TCCATGTTGT	IDT	N/A
TaqMan assays for ddPCR	Thermo Fisher Scientific	Assay IDs: See Table S4

**Software and algorithms**		

BWA (Burrows-Wheeler Aligner) v0.7.17	Li and Durbin^112^	http://bio-bwa.sourceforge.net/
deepTools2 (bamCoverage) v3.5.0	Ramirez et al.^113^	https://deeptools.readthedocs.io/
featureCounts v1.6.5	Liao et al.^114^	http://subread.sourceforge.net/
limma (removeBatchEffect) v3.38.0	Ritchie et al.^115^	https://bioconductor.org/packages/limma
DESeq2 v1.30.0	Love et al.^116^	https://bioconductor.org/packages/DESeq2
edgeR (differential exon usage) v3.32	Robinson et al.^117^	https://bioconductor.org/packages/edgeR
g:Profiler (R package) v0.2.4	Raudvere et al.^118^	https://biit.cs.ut.ee/gprofiler/
MACS2 (peak calling) v2.2.7.1	Zhang et al.^119^	https://github.com/macs3-project/MACS
SAMtools v1.14	Li et al.^120^	http://www.htslib.org/
BEDTools (coverageBed) v v2.30.0	Quinlan and Hall^121^	https://bedtools.readthedocs.io/
CHIPseeker (annotatePeak) v 1.28.1	Yu et al.^122^	https://bioconductor.org/packages/ChIPseeker
Bowtie 2 v2.4.4	Langmead and Salzberg^123^	http://bowtie-bio.sourceforge.net/bowtie2/
SEACR (peak calling for CUT&RUN)	Meers et al.^124^	https://github.com/FredHutch/SEACR
trim_galore v0.6.7	Babraham Bioinformatics	https://www.bioinformatics.babraham.ac.uk/projects/trim_galore/
motifbreakR v2.8.0	Coetzee et al.^125^	https://bioconductor.org/packages/motifbreakR
CellProfiler v3.0	McQuin et al.^126^	https://cellprofiler.org/
uncertainties v3.2.0 (Python package)	lmfit GitHub organization	https://pythonhosted.org/uncertainties/
Analysis scripts (all custom code)	This paper	Zenodo: https://doi.org/10.5281/zenodo.19360201
R statistical software v4.3.0	R Core Team	https://www.r-project.org/
Python v3.11	Python Software Foundation	https://www.python.org/

**Other**		

BD LSRll flow cytometer	BD Biosciences	N/A
QX200 Droplet Generator	Biorad	Cat# 1864002
QX200 Droplet Reader	Biorad	Cat# 1864003
Luminex MAGPIX instrument	Luminex	N/A
Lonza 4D-Nucleofector (program CA137)	Lonza	N/A
Illumina MiSeq (amplicon genotyping)	Illumina	N/A
Illumina HiSeq (RNAseq)	Illumina	N/A
Illumina Novaseq 6000 (ATACseq, CUT&RUN)	Illumina	N/A
Transwell inserts (PET, 5 μm pores)	Sarstedt	Cat# 83.3932.500
EVOS FL Auto automated microscope	Thermo Fisher Scientific	N/A

### Experimental model and study participant details

#### Human induced pluripotent stem cell (hiPSC) line

KOLF2_C1 (https://hpscreg.eu/cell-line/WTSIi018-B-1) is a male human induced pluripotent stem cell (hiPSC) line derived as part of the HipSci project. Derivation was approved by the NRES Research Ethics Committee (Cambridgeshire 1 NRES REC ref. 09/H0304/77, HMDMC 14/013) and informed consent was obtained from the donor. The KOLF2 line was subcloned from a single cell to ensure population homogeneity to give KOLF2_C1. The parental KOLF2_C1 line is homozygous for the T (non-risk) allele at rs28834970. This study exclusively uses cells derived from a single male donor; the influence of sex on the results was therefore not analyzed and represents a limitation to the generalisability of the findings.

KOLF2_C1 cells were cultured in TeSR E8 medium (StemCell Technologies) on Synthemax-coated surfaces (Corning; final concentration 2 μg/cm^2^) and routinely passaged 1:10 every 5 days using Gentle Cell Dissociation Reagent (StemCell Technologies). Cells were maintained at 37°C in a humidified atmosphere with 5% CO2. The KOLF2_C1 cell line was authenticated by whole genome sequencing but edited derivatives were not independently authenticated beyond genotyping for the rs28834970 variant. All cell lines tested negative for mycoplasma. Cell line sex: male.

#### Engineered isogenic hiPSC clones

Three homozygous C/C clones (C8, C11, D3) and two T/T clones (unedited parental KOLF2_C1 and a clonally expanded unedited clone B11) were used for all downstream differentiation experiments. All clones are male. Editing and clonal selection procedures are described in method details below.

#### hiPSC-derived macrophages and microglia

Macrophages and microglia were derived *in vitro* from the isogenic hiPSC clones by directed differentiation (see method details). Differentiated cells are non-dividing and were not authenticated separately from their parental hiPSC lines. All differentiation experiments were performed in three independent biological replicates per clone. Cells were maintained at 37°C, 5% CO2 throughout differentiation.

### Method details

#### Engineering the rs28834970 C/C allele in hiPSCs

The C/C risk allele at rs28834970 was introduced into KOLF2_C1 hiPSCs by nucleofection of a ribonucleoprotein (RNP) complex containing eSpCas9 and a chemically modified synthetic guide RNA, together with a single-stranded oligodeoxynucleotide (ssODN) repair template, following the protocol of Bruntraeger et al.

eSpCas9 was expressed from Escherichia coli using a His-tag purification strategy. KOLF2_C1 cells were cultured on Synthemax (5 μg/cm^2^) in TeSR E8 medium prior to nucleofection. eSpCas9 (5 μL, 20 μg) was mixed with the chemically modified guide RNA (see key resources table for sequence: AGTGGAATTGTACAACACTG; Synthego; 5 μL, 225 pmol) at room temperature for 20 min to allow RNP complex assembly. The ssODN repair template (5 μL, 500 pmol; sequence: CTGGCAAGACTAATCTACTTTCTATTTTTATGGATCTGCCTTTTCTGGTCATTCCATATAAGTGGAATTGCACAACACTGTGGCCTTTCGCGACGGCTGCTTTCACTTAGCACAATGTTTTGAAACTTCCTCCATGTTGT) was then added immediately before nucleofection.

Cells were detached with Accutase (StemCell Technologies) and dissociated to a single-cell suspension. 1 × 10^6^ cells were resuspended in P3 buffer (Lonza), combined with the RNP/template mix, and nucleofected using a Lonza 4D-Nucleofector with program CA137. Cells were seeded onto a 10 cm Synthemax-coated dish (5 μg/cm^2^) in TeSR E8 supplemented with CloneR (StemCell Technologies), expanded to ∼80% confluency, then dissociated and sub-cloned by plating 5,000 cells on a fresh 10 cm dish.

Individual colonies were picked for genotyping by high-throughput amplicon sequencing on an Illumina MiSeq instrument using a primer pair spanning rs28834970. Final selected clones were confirmed by Sanger sequencing. Three independent homozygous C/C clones (C8, C11, D3) and one T/T unedited clone (B11) were selected alongside the parental KOLF2_C1 line for downstream experiments.

#### hiPSC differentiation to macrophages

iPSCs were differentiated to macrophages following an embryoid body (EB)-based protocol as previously described. Briefly, KOLF2_C1 cells were cultured in Essential E8 medium (Gibco) on Vitronectin (Gibco, 100×) and transferred onto a feeder layer of Mouse Embryonic Fibroblasts (MEFs; Merck) using Gentle Cell Dissociation Reagent (StemCell Technologies). hiPSCs were cultured on MEFs in hiPSC base medium (Advanced DMEM F12 [Gibco], KnockOut Serum Replacement [Gibco], 2 mM Glutamax [Gibco], 100 U/ml penicillin/streptomycin [Gibco], 0.055 mM 2-mercaptoethanol [Sigma], 4 ng/mL recombinant human FGF [R&D Systems]) until colonies were sufficiently large.

hiPSC colonies were lifted with a 1:1 solution of Collagenase type IV and Dispase type II (ThermoFisher Scientific) for 15 min at 37°C and transferred to 10 cm low-adherent dishes in hiPSC base medium without FGF for EB formation. After 5 days, EBs were plated onto 0.1% gelatin-coated 10 cm dishes in EB medium (X-Vivo 15 [Lonza], 2 mM Glutamax, 100 U/ml penicillin/streptomycin, 0.055 mM β-mercaptoethanol, 50 ng/mL rhM-CSF [R&D Systems], 25 ng/mL rhIL-3 [R&D Systems]). Macrophage precursor cells were harvested from dishes every 6–7 days for up to 3–4 months. Terminal differentiation was achieved by plating 15,000 cells/cm^2^ in macrophage medium (RPMI 1640 [Gibco], 10% FBS [Thermo Fisher Scientific], 2 mM Glutamax, 100 U/ml penicillin/streptomycin, 100 ng/mL rhM-CSF) for 7 days at 37°C.

#### hiPSC differentiation to microglia

hiPSCs were differentiated to microglia following a previously described protocol. KOLF2_C1 cells were cultured in Essential E8 medium (Gibco) on Synthemax (5 μg/cm^2^). Two days after passaging, cells were detached with Accutase, dissociated to single cells, and 10,000 hiPSCs were seeded per well of a 96-well plate in EB medium (Essential E8, 10 μM Y-27632 [StemCell Technologies], 50 ng/mL rhBMP-4 [Peprotech], 20 ng/mL rhSCF [Peprotech], 50 ng/mL rhVEGF [Peprotech]). Plates were centrifuged at 300×g for 3 min and incubated for 4–5 days for EB formation.

EBs were transferred to 0.1% gelatin-coated tissue culture dishes in Microglia Precursor medium (X-Vivo 15 [Lonza], 2 mM Glutamax, 100 U/ml penicillin/streptomycin, 0.055 mM β-mercaptoethanol, 100 ng/mL rhM-CSF [Peprotech], 25 ng/mL rhIL-3 [Cell Guidance Systems]) at 37°C. Microglia precursor cells were harvested every 6–7 days for up to 3 months. Terminal differentiation was achieved by plating precursors at 180,000 cells/cm^2^ in Microglia medium (RPMI 1640 [Gibco], 10% FBS [Thermo Fisher Scientific], 2 mM Glutamax, 100 U/ml penicillin/streptomycin, 10 ng/mL rhGM-CSF [Peprotech], 100 ng/mL rhIL-34 [Peprotech]) for 7 days at 37°C.

#### Fluorescence-activated cell sorting (FACS)

Microglia or macrophages were detached using Accutase (StemCell Technologies) and washed twice in phosphate-buffered saline (PBS). 1 × 10^5^ cells were blocked with Human TruStain FcX (Biolegend) for 30 min at 4°C in the dark. Microglia were stained with 5 μL each of PE-anti human CD45 (clone HI30; Biolegend) and APC-anti human CD11b (clone ICRF44; Biolegend) for 30 min at 4°C. Macrophages were stained with 5 μL each of APC/Cy7-anti human CD16 (clone 3G8; Biolegend), APC-anti human CD206 (BD Biosciences) or PE-anti human CD14 (BD Biosciences). Corresponding isotype controls were used at 0.2 mg/mL: APC-mouse IgG1 k, PE-mouse IgG2A, and APC/Cy7-mouse IgG1 k (all BD Biosciences). Cells were washed three times in FACS buffer (5% FBS in D-PBS), resuspended in 200 μL DAPI solution (1 μg/mL in PBS), and analyzed on a BD LSRll instrument. Differences between T/T and C/C genotypes were assessed with an unpaired *t* test.

#### RNA extraction and reverse transcription

Cells were washed in PBS and lysed directly on the plate with TRI Reagent (Zymo). RNA was extracted using the Direct-zol RNA Miniprep kit (Zymo) with on-column DNase digestion, according to the manufacturer’s protocol. RNA was eluted in 50 μL DNase/RNase-free water. 1 μg of RNA was reverse transcribed to cDNA using SuperScript IV Reverse Transcriptase (Thermo Fisher Scientific) with random hexamers, according to the manufacturer’s protocol.

#### RNA sequencing

Transcriptome libraries for hiPSC-derived macrophages and microglia were generated using the Illumina TruSeq Stranded RNAseq kit (polyA selection). All samples were sequenced on an Illumina HiSeq instrument to a depth of approximately 50 million mapped reads per sample.

Reads were mapped to the human reference genome (GRCh38/hg38) using BWA. Coverage tracks were generated with bamCoverage (deepTools2) normalised by Counts per Million (CPM) and visualised on the UCSC Genome Browser. Read counts per transcript were quantified with featureCounts and used to calculate Transcripts per Million (TPM) using a custom R script. For macrophage samples, TPMs were corrected for batch effects using limmaremoveBatchEffect. For microglia, all samples were differentiated and sequenced in a single batch. Differential gene expression between C/C and T/T genotypes was analyzed using raw counts with DESeq2. Principal component analysis plots were generated from variance-stabilising transformation (vst)-transformed counts. Differential exon usage for PTK2B was assessed using edgeR. Gene ontology (GO) enrichment analysis of differentially expressed genes was performed using the g:Profiler R package. Thresholds for differential expression were log2FC > 0.5 or < −0.5 and adjusted *p* < 0.05.

#### ATACseq

hiPSC-derived macrophages and microglia were detached using a solution of 4 mg/mL Lidocaine hydrochloride monohydrate (Sigma) and 5 mM EDTA (ThermoFisher Scientific) for 15 min at 37°C. ATACseq was performed as described by Buenrostro et al. Cells were lysed in sucrose buffer (10 mM Tris-Cl pH 7.5, 3 mM CaCl2, 2 mM MgCl2, 0.32 M sucrose) and permeabilised in 0.5% Triton X-100 (Sigma). Nuclei were pelleted at 450×g for 5 min at 4°C. Tagmentation was performed with Illumina Tagment DNA TDE1 Enzyme and Buffer Kit using 5 μL TDE1 enzyme per 100,000 cells for 30 min at 37°C. Reactions were stopped with MinElute buffer PB (Qiagen) and purified DNA was eluted in 10 μL EB. Libraries were amplified by PCR with dual-indexed Illumina adapters, gel-purified, and sequenced on an Illumina Novaseq 6000 to approximately 30 million reads per sample.

Reads were mapped to GRCh38/hg38 with BWA and duplicate reads removed with Picard MarkDuplicates. Coverage tracks were normalised by CPM using bamCoverage (deepTools2). All samples were subsampled to the read depth of the lowest-coverage sample using samtools view. Peaks were called with MACS2 (flags: --shift −100 --extsize 200). A merged peak file was generated from all samples and read counts under each peak were quantified in all samples using bedtools coverageBed. Differential chromatin accessibility between genotypes was analyzed with DESeq2. Peaks were annotated to genes using CHIPseekerannotatePeak.

#### CUT&RUN assay

Microglia were detached with 4 mg/mL Lidocaine hydrochloride monohydrate and 5 mM EDTA for 15 min at 37°C. CUT&RUN for CEBPβ was performed using the CUTANA CUT&RUN Kit (EpiCypher) following the manufacturer’s instructions with optimised permeabilisation using 0.01% Digitonin. 400,000 microglia were used per reaction. 1 μg of C/EBP beta Antibody (H-7; Santa Cruz Biotechnology, Cat# sc-7962) was used per sample; a parallel negative control was treated with 1 μL of CUTANA Rabbit IgG CUT&RUN Negative Control Antibody (EpiCypher). 0.5 ng of E. coli Spike-in DNA (EpiCypher) was added to each sample for normalisation of sequencing depth and experimental variability. Approximately 5 ng of purified CUT&RUN-enriched DNA was used to prepare libraries with NEBNext Ultra II DNA Library Prep Kit and NEBNext Multiplex Oligos for Illumina (Dual Index Primers Set 1; NEB). Libraries were sequenced 150 bp paired-end on an Illumina Novaseq 6000 to approximately 15 million reads per sample.

Reads were trimmed to remove Illumina adapters using trim_galore and mapped to GRCh38/hg38 using Bowtie 2. All files were subsampled to the read depth of the lowest-coverage sample with samtools. Trimmed reads were also mapped to the E. coli genome using Bowtie 2 and human-genome-mapped reads were normalised to spike-in E. coli DNA using a published calibration script (https://github.com/Henikoff/Cut-and-Run/blob/master/spike_in_calibration.csh) integrated into a custom bash script (https://github.com/ericabello/PTK2B_rs28834970/blob/main/Cut_Run_CEBPb_micro/cmds_CutRun_CEBPb_micro.sh). Coverage tracks were generated as bigwig files from E. coli-normalised bedgraph files using UCSC bedGraphToBigWig. Peaks were called in all CEBPβ antibody samples with SEACR using the IgG negative control to define the signal threshold. A merged peak file was created and reads under each peak quantified with bedtools coverageBed. Differential CEBPβ binding between genotypes was assessed with DESeq2.

#### Digital droplet qPCR (ddPCR)

50 ng of cDNA per sample was combined with 10 μL ddPCR Supermix for Probes (no dUTP; Biorad), 1 μL of FAM-tagged TaqMan assay for the gene of interest, and 1 μL of VIC-tagged TaqMan assay for the appropriate housekeeping gene. Housekeeping genes were selected to match the expression level of the target gene in each cell type. The full list of TaqMan assays (ThermoFisher Scientific) used for macrophages and microglia is provided in method details (see Table S4). Droplets were generated with a QX200 Droplet Generator (Biorad) and transferred to 96-well plates. Thermal cycling was performed as follows: 95°C for 10 min; 40 cycles of 95°C for 30 s and 60°C for 1 min; 98°C for 10 min. FAM and VIC fluorescence was measured with a QX200 Droplet Reader (Biorad). Poisson statistics were used to determine absolute copy numbers per μL for both target and housekeeping genes. Target gene copies were normalised to the housekeeping gene for each reaction. Differences between T/T and C/C genotypes were tested with an unpaired *t* test followed by Benjamini-Hochberg multiple testing correction.

#### Microglia stimulation

hiPSC-derived microglia at day 6 of differentiation were stimulated with recombinant human IFNγ (R&D Systems; 200 ng/mL) or Lipopolysaccharide (LPS; Invitrogen; 100 ng/mL) for 24 h at 37°C. For the migration assay, microglia at day 7 were stimulated with IFNγ (100 ng/mL) for 72 h.

#### Luminex multiplex chemokine assay

Following stimulation, culture plates were centrifuged at 4,000 rpm for 20 min at 4°C and conditioned media were collected. Media were diluted 1:20 (unstimulated) or 1:250 (stimulated) for analysis. Luminex assays were performed using the ProcartaPlex Human Basic Kit (Invitrogen) with a custom panel of 13 ProcartaPlex Human Simplex analyte beads (Invitrogen), according to the manufacturer’s instructions. Plates were read on a Luminex MAGPIX instrument. Cells with T/T and C/C alleles were always run on the same plate to allow direct comparison. Differences in chemokine concentration (pg/mL) between genotypes were tested with an unpaired *t* test followed by Benjamini-Hochberg correction. Propagation of error for log fold-change values was calculated with the Python uncertainties package (https://pythonhosted.org/uncertainties/).

#### Microglia migration (transwell) assay

At day 10 of differentiation (following 72-h IFNγ stimulation where applicable), microglia were detached with 4 mg/mL Lidocaine hydrochloride monohydrate and 5 mM EDTA (Gibco) and 5.5 × 10^4^ cells in 100 μL macrophage medium were seeded onto transwell inserts (PET membrane, 5 μm pore size; Sarstedt) in a 24-well plate. After 15 min to allow cell settling, 600 μL macrophage medium containing 3 nM recombinant human C5a was added to the lower chamber. Migration was allowed to proceed for 10 h at 37°C. Transwells were rinsed in PBS, fixed with 4% paraformaldehyde for 20 min at room temperature, and both sides of the membrane stained with NucBlue (Invitrogen). The intact transwell was imaged first (“top image”) on an EVOS FL Auto automated microscope (Thermo Fisher), using a whole-well tiling acquisition. Cells on the upper surface were removed by swabbing with a cotton bud, and the transwell was imaged again (“bottom image”) under identical settings. Cell counting was performed with CellProfiler 3.0. The percentage of migrated cells was calculated as: (cells in bottom image)/(cells in top image) × 100. Stimulations were performed in triplicate; the mean per clone across wells was used for analysis. Genotype differences were assessed with an unpaired *t* test.

### Quantification and statistical analysis

Statistical details for all experiments are reported in the figure legends. Briefly, an unpaired two-tailed *t* test was used to assess differences between cells with the T/T and C/C allele at rs28834970 in FACS analysis, ddPCR, Luminex assay, and migration assays. For ddPCR and Luminex assays, multiple testing correction was applied using the Benjamini-Hochberg procedure. For RNAseq and ATACseq analyses, differential expression and accessibility were determined using DESeq2,^116^ which employs a negative binomial model with shrinkage estimation of dispersion and a Wald test to identify differential expression. For each experiment, n refers to the number of independent biological differentiation replicates (*n* = 3 per clone across five clones unless stated otherwise). Data are presented as mean ± SEM unless otherwise stated. Statistical significance was defined as adjusted *p* < 0.05. Datapoints were not excluded from analyses.

## Supplemental information

Document S1. Figures S1–S5

Table S1. ATACseq analysis in macrophages and microglia, related to Figure 2

Table S2. CEBPβ CUT&RUN analysis in microglia, related to Figure 2

Table S3. RNAseq analysis in macrophages and microglia, related to Figures 2 and 3

Table S4. ddqPCR assay in macrophages and microglia (TaqMan assay IDs and results), related to Figures 2 and 3

Table S5. Luminex assay in microglia, related to Figure 4

Table S6. ENA accession numbers for the datasets, related to Figures 2 and 3
