# Supplementary material for: An Alzheimer’s disease-associated common regulatory variant in a PTK2B intron alters microglial function
Source: iScience. 2026 Apr 9;29(5):115688. doi: 10.1016/j.isci.2026.115688 (PMC13187524; doi:10.1016/j.isci.2026.115688)
Supplement: Document S1. Figures S1–S5 [file mmc1.pdf]

iScience, Volume *29*

Supplemental information

An Alzheimer's disease-associated common regulatory variant in a *PTK2B* intron alters microglial function

Erica Bello, Kathleen Long, Sho Iwama, Juliette Steer, Sarah E. Cooper, Kaur Alasoo, Natsuhiko Kumasaka, Jeremy Schwartzentruber, Nikolaos I. Panousis, and Andrew R. Bassett

Supplementary Figures

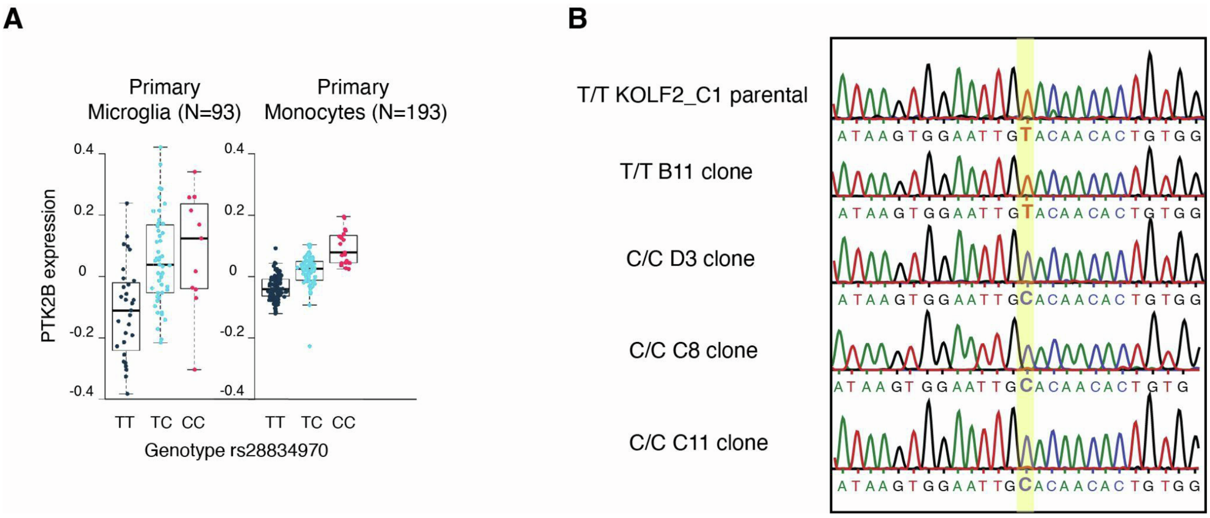

Figure S1: Characterisation of the rs28834970 variant in PTK2B

A) Boxplots showing the expression of the *PTK2B* gene stratified by the rs28834970 genotype in primary microglia and primary monocytes from[25]. The y-axis shows normalised expression levels (log TPM value) and each dot on the box shows the expression level of a single sample. B) Sanger sequencing tracks of an amplicon around the rs28834970 variant in wild-type T/T KOLF2_C1 hiPSC clones (the KOLF2_C1 parental line and B11 T/T clone which has been through the editing and clonal selection process) and engineered homozygous C/C KOLF2_C1 clones (D3, C8 and C11). The rs28834970 variant is highlighted in yellow. Related to Figure 1.

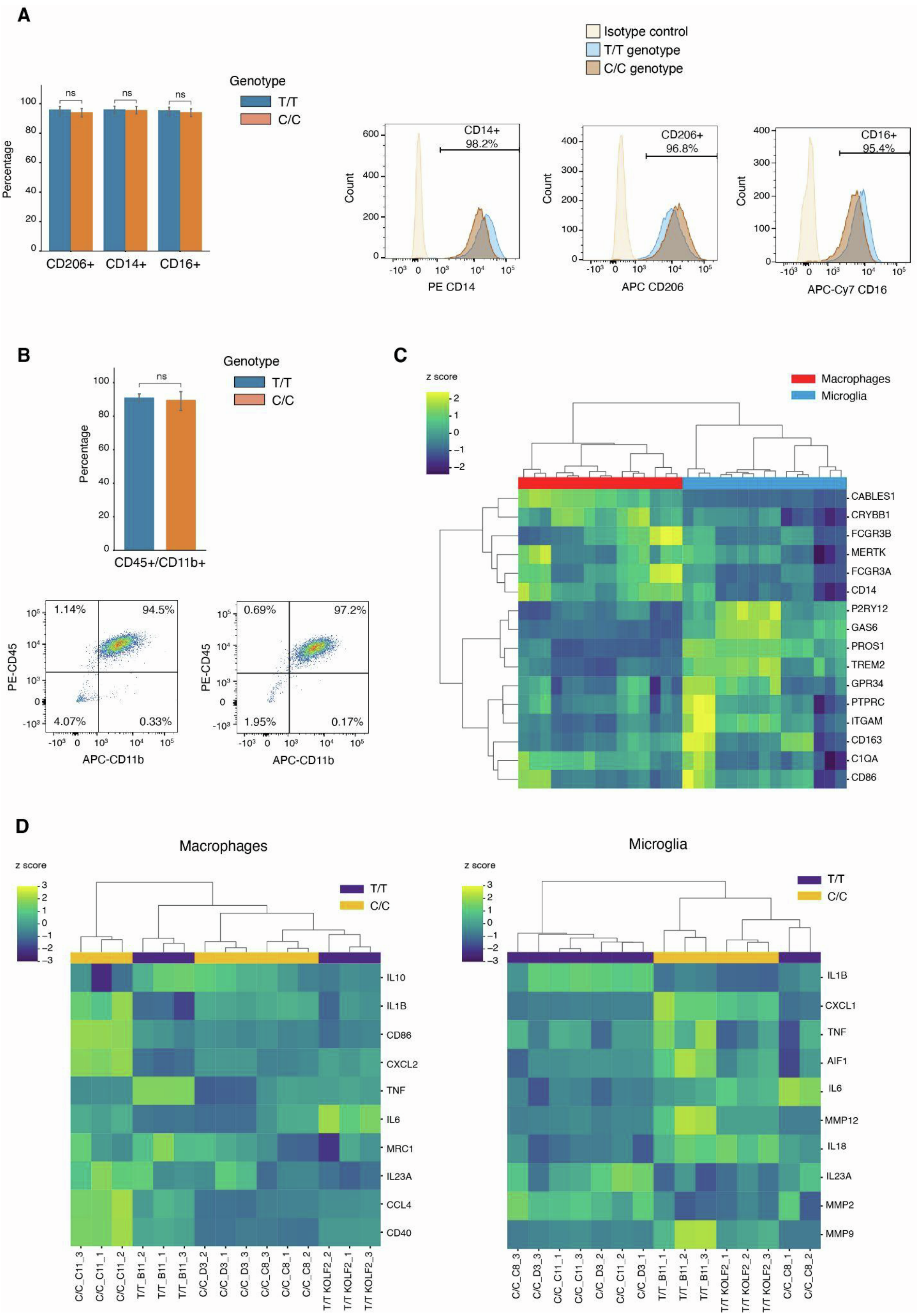

Figure S2: Characterisation of differentiation of hiPSC-derived macrophages and microglia
A) Bar graph showing the percentage of macrophages harbouring either the T/T or C/C allele positive for each marker (left). Results are shown as the mean ± SEM of independent

biological replicates (n=3). ns= non-significant, unpaired t-test. Representative histograms from FACS analysis of single-stained macrophage markers CD14, CD16 and CD206 in hiPSC-derived macrophages with either T/T or C/C allele at rs28834970 (right). B) Bar graph showing the percentage of CD11b+CD45+ microglia with the T/T or C/C allele (top). Results are shown as the mean ± SEM of independent biological replicates (n=3). ns= non-significant, unpaired t-test. Representative scatterplots from FACS analysis of microglia markers CD11b and CD45 in hiPSC-derived microglia harbouring the T/T or C/C allele at rs28834970 (bottom). C) Heatmap showing the relative expression of microglia and macrophage markers[75,101,102] in hiPSC-derived macrophages (red) and microglia (blue) harbouring the T/T or C/C allele at rs28834970. D) Heatmap showing the relative expression of activation markers[103,104] of macrophages (left) and microglia (right) in the corresponding hiPSC-derived cell type harbouring either the T/T (orange) or the C/C (purple) allele at rs28834970. Related to Figure 3.

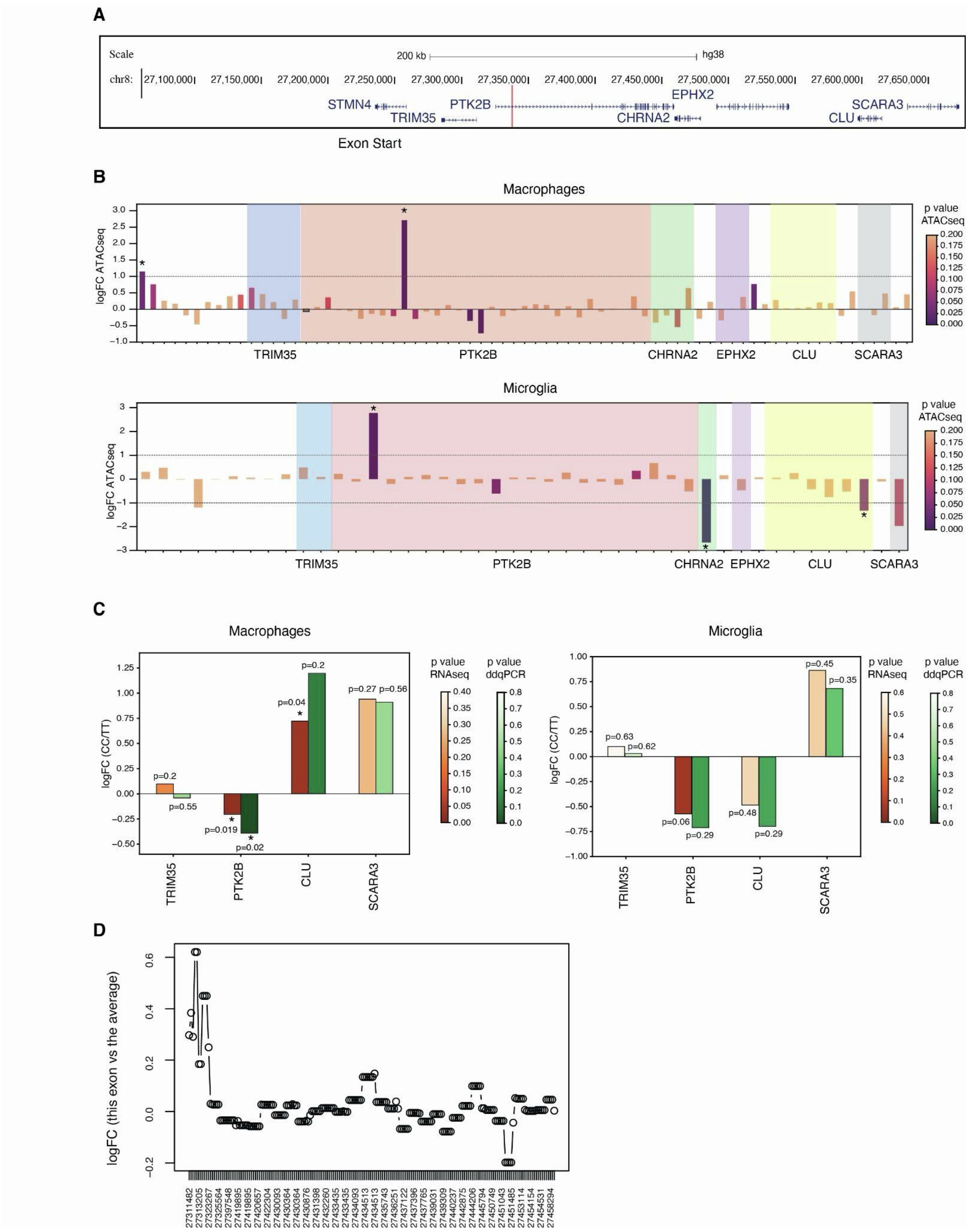

Figure S3: Effects of the rs28834970 variant on the surrounding genomic region

A) Track from UCSC Genome Browser (Human GRCh38/hg38) showing RefSeq genes in a 600 kb window around the rs28834970 SNP (highlighted in red). B) Bar graph showing logFC of chromatin accessibility between macrophages (top) and microglia (bottom) harbouring the C/C versus the T/T allele for all ATAC-seq peaks identified in the 600kb window around rs28834970. The colour of the bars indicates the p value and peaks (bars) located in the same gene are highlighted in the same colour. The asterisks denote peaks reaching significance

(logFC>+-1 and $p<0.05$). C) Bar graph showing the log fold change (FC) of expression of the genes located in the window around rs28834970 in macrophages (left) and microglia (right) with the C/C versus the T/T allele, measured by RNAseq (red) and ddqPCR (green). ddqPCR n=3 * $p<0.05$, **$p<0.01$ unpaired t-test. D) Plot showing the results of the analysis of differential exon usage between microglia harbouring the C/C and the T/T allele at rs28834970. The relative logFC of expression of each exon of *PTK2B* is the difference between the exon's logFC and the overall logFC for the gene. None of the *PTK2B* exons (shown as dots) were significantly changed. Related to Figure 3.

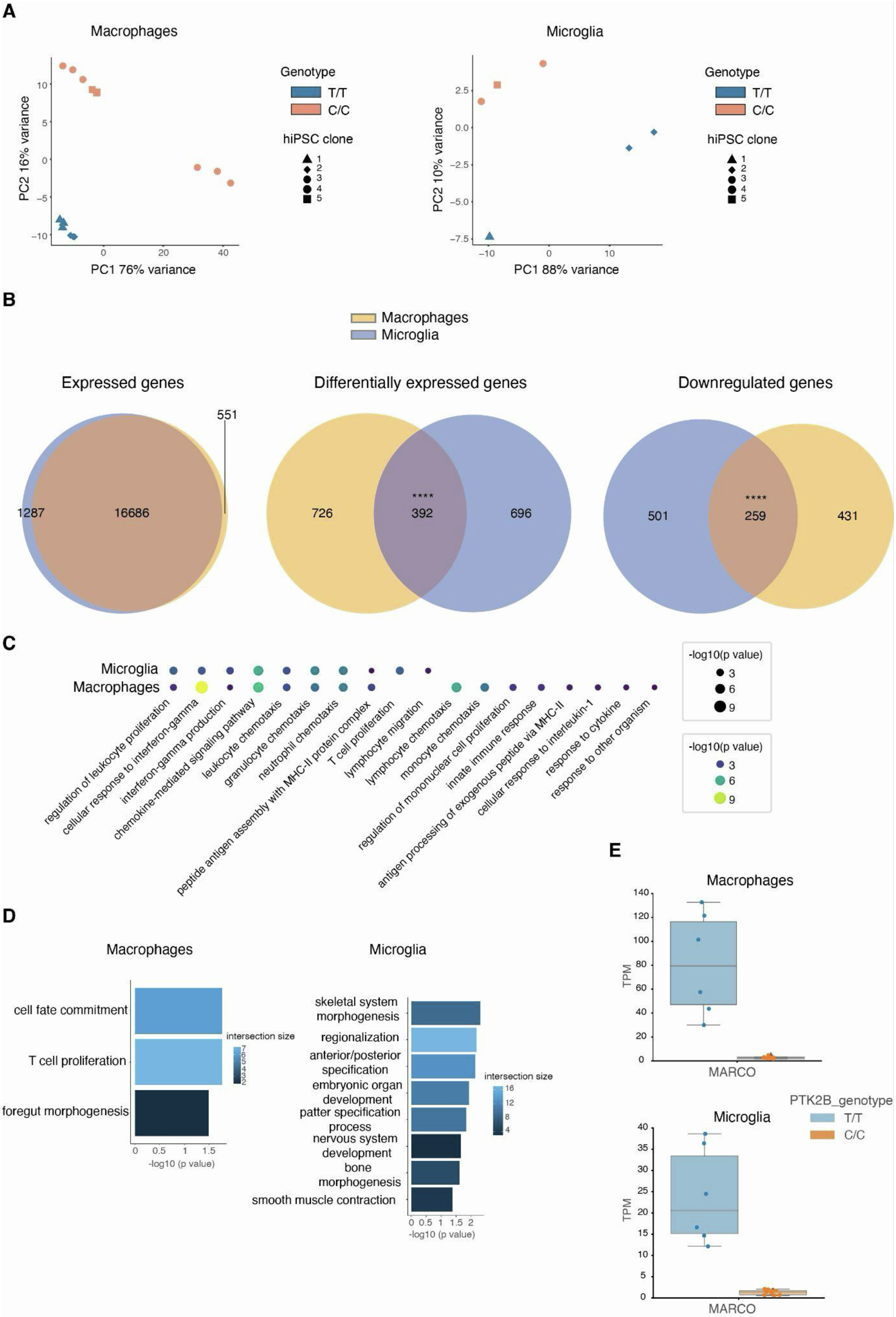

Figure S4: Characterisation of the effects of rs28834970 on chromatin accessibility and gene expression in hiPSC-derived macrophages and microglia

A) Principal component analysis (PCA) of accessible regions across the genome of macrophages (left) and microglia (right) differentiated from two hiPSC clones with the T/T allele and three clones with the C/C allele, measured by ATAC-seq.
B) Venn diagrams showing overlaps between microglia and macrophages of all expressed genes (left), all differentially expressed genes (middle) and downregulated genes (logFC<-0.5 and p<0.05) (right), measured by RNA-seq. Statistical significance of number of overlaps was calculated using Chi-square test with Yates' correction. ****p<0.0001
C) Top pathways identified in GO analysis of downregulated genes in microglia and macrophages. Colour and size of the dots represents the -log10 of the p-value.
D) Results of GO analysis of upregulated genes (logFC>0.5 and p<0.05) in macrophages (left) and microglia (right) with the C/C versus the T/T allele at rs28834970.
E) Expression of *MARCO* in macrophages (top) and microglia (bottom) harbouring the T/T or C/C allele at rs28834970, measured by RNA-seq. Cells were differentiated from two hiPSC clones with the T/T allele and three clones with the C/C allele. TPM= transcripts per million, differential expression analysis performed using DESeq2. Related to Figure 3.

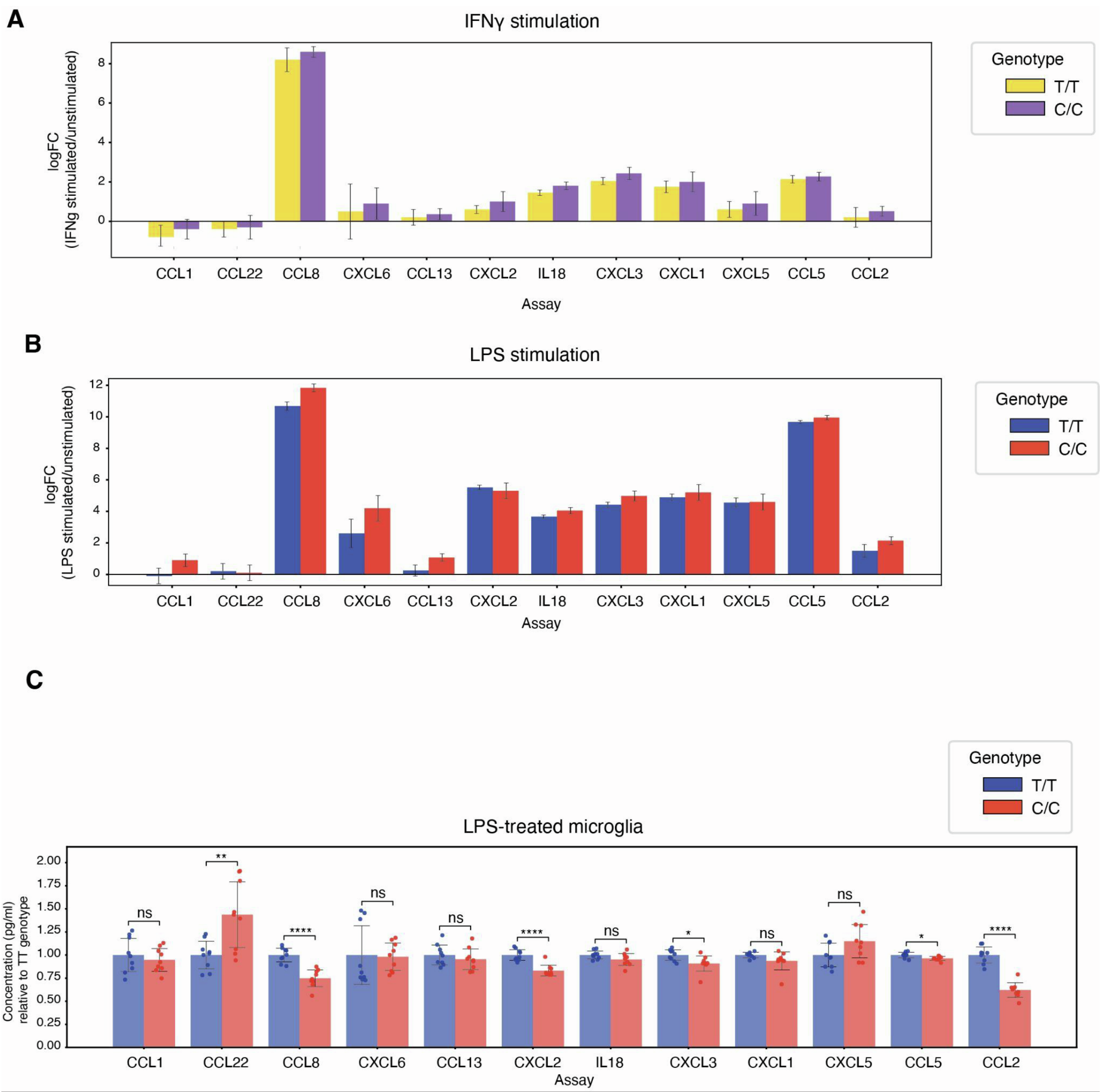

Figure S5: The rs28834970 variant causes changes in chemokine production hiPSC-derived microglia

A) Bar graph showing logFC of chemokine concentration between unstimulated microglia and microglia stimulated with IFNγ. Yellow bars represent microglia with the T/T allele and purple bars show logFC of microglia with the C/C allele. Results are shown as the mean ± propagation of error of independent biological replicates (n=3). B) Bar graph showing logFC of chemokine concentration between unstimulated microglia and microglia stimulated with LPS. Blue bars represent microglia with the T/T allele and red bars show logFC of microglia with the C/C allele. Results are shown as the mean ± propagation of error of independent biological replicates (n=3). C) Concentration of downregulated chemokines in microglia with the C/C allele at rs28834970 relative to the concentration in cells with the T/T allele, stimulated with LPS. Bars represent the mean ± SEM of independent biological replicates (n=3) of each of the five differentiated hiPSC clones and dots represent technical replicates per clone. ns= non-significant, * p<0.05, **p<0.01, ***p<0.001, ****p<0.0001, unpaired t-test. Related to Figure 5.

Supplementary references

1. Young, A. M. H. *et al.* A map of transcriptional heterogeneity and regulatory variation in human microglia. *Nat. Genet.* **53**, (2021).
2. Brownjohn, P. W. *et al.* Functional Studies of Missense TREM2 Mutations in Human Stem Cell-Derived Microglia. *Stem Cell Reports* **10**, 1294–1307 (2018).
3. Haenseler, W. *et al.* A Highly Efficient Human Pluripotent Stem Cell Microglia Model Displays a Neuronal-Co-culture-Specific Expression Profile and Inflammatory Response. *Stem Cell Reports* **8**, 1727–1742 (2017).
4. Vaughan-Jackson, A. *et al.* Differentiation of human induced pluripotent stem cells to authentic macrophages using a defined, serum-free, open-source medium. *Stem Cell Reports* **16**, 3093 (2021).
5. Jurga, A. M., Paleczna, M. & Kuter, K. Z. Overview of General and Discriminating Markers of Differential Microglia Phenotypes. *Front. Cell. Neurosci.* **14**, 544457 (2020).
6. Murray, P. J. *et al.* Macrophage activation and polarization: nomenclature and experimental guidelines. *Immunity* **41**, (2014).
